# Emergence of polarized opinions from free association networks

**DOI:** 10.3758/s13428-018-1090-z

**Published:** 2018-08-09

**Authors:** Bálint File, Zsolt Keczer, Anna Vancsó, Beáta Bőthe, István Tóth-Király, Márton Hunyadi, Adrienn Ujhelyi, István Ulbert, Júlia Góth, Gábor Orosz

**Affiliations:** 1 Institute of Cognitive Neuroscience and Psychology, Research Centre for Natural Sciences, Hungarian Academy of Sciences, Magyar Tudósok Körútja 2., Budapest, H-1117 Hungary; 2 Faculty of Information Technology and Bionics, Pázmány Péter Catholic University, Budapest, Hungary; 3 Wigner Research Centre for Physics, Hungarian Academy of Sciences, Budapest, Hungary; 4 Doctoral School of Psychology, Eötvös Loránd University, Budapest, Hungary; 5 Faculty of Education and Psychology, Institute of Psychology, Eötvös Loránd University, Budapest, Hungary; 6 Doctoral school of Sociology, Corvinus University of Budapest, Budapest, Hungary; 7 Department of Psychology, Stanford University, Stanford, CA Hungary

**Keywords:** Association, Polarized opinions, Asylum seekers, Opinion network

## Abstract

We developed a method that can identify polarized public opinions by finding modules in a network of statistically related free word associations. Associations to the cue “migrant” were collected from two independent and comprehensive samples in Hungary (*N*_1_ = 505, *N*_2_ = 505). The co-occurrence-based relations of the free word associations reflected emotional similarity, and the modules of the association network were validated with well-established measures. The positive pole of the associations was gathered around the concept of “Refugees” who need help, whereas the negative pole associated asylum seekers with “Violence.” The results were relatively consistent in the two independent samples. We demonstrated that analyzing the modular organization of association networks can be a tool for identifying the most important dimensions of public opinion about a relevant social issue without using predefined constructs.

**Electronic supplementary material:**

The online version of this article (10.3758/s13428-018-1090-z) contains supplementary material, which is available to authorized users.

In the present study, we aimed to use one socially prominent issue as a cue (asylum seekers, labeled as “migrants”) to capture opinions shared by a social group (Hungarians) (Abric, 1993; Moscovici, 1984; Wagner et al., 1999). As a measure of public opinion, the free association method can be viewed as a semistructured alternative between traditional questionnaires, producing highly structured data, and Web-mining algorithms, collecting large quantities of unstructured data. Hence, the free association method can overcome the predefined scope of questionnaires (Bansak, Hainmueller, & Hangartner, 2016), since respondents can freely express their opinion, yet it has the advantage of representative samples and fast data processing, as opposed to several Web-mining methods (Lazer, Kennedy, King, & Vespignani, 2014). Traditionally, free association analysis has focused on consensual meaning (i.e., the most frequent words and rankings) regarding a social object (Abric, 1993; Moscovici, 1984; Wagner et al., 1999) and has not focused on the polarization of opinions (Bradley, Mogg, & Williams, 1995; Halberstadt, Niedenthal, & Kushner, 1995; Joffe & Elsey, 2014; Niedenthal, Halberstadt, & Innes-Ker, 1999).

Different prior word association methods were introduced in order to distinguish the stable and recurrent associations from peripheral ones. Szalay and Brent (1967) developed the associative group analysis approach of free associations. In this method, the early associations in a continued association task were found to have a high probability of being produced again during a retest. Previous studies in social representation theory (Abric, 1993; Wagner, Valencia, & Elejabarrieta, 1996) have argued that frequent associations are temporally stable and they refer to the consensual meaning regarding a given social object (a.k.a. the central core of the social representation). Alternatively, Kinsella and her coworkers (Kinsella, Ritchie, & Igou, 2015) used the prototype analysis of free associations, in which most frequent associations (above a threshold) are considered as the consensual prototype of the social object in the perception of the social group.

Despite of the stable core of the representations, social issues can trigger opposite emotions, interpretations, attitudes, ideas and beliefs in a society, which can yield a polarized structure of public opinions. With sufficient data, it is possible to organize free associations not only along a core–periphery dimension, but to identify a more detailed structure with multiple major frames of interpretation in a society. Prior research used the available up-to-date technology to analyze free associations in relation to ideology (Szalay, Kelly, & Moon, 1972) and attitude measurement (Szalay, Windle, & Lysne, 1970). Furthermore, Szalay and Deese (1978) provided an extensive summary of their pioneering factor analytic method for word associations. Apart from these works, to our best knowledge, no recent data-driven studies focused on the polarization of opinions with free associations. Therefore, we aimed to fill this methodological gap.

## Method demonstration: Public opinion of “migrants”

We aimed to demonstrate our method on public opinions about the recent “migration crisis,” which had a significant political and social effect in many European countries, including Hungary. The increased number of asylum seekers made migration one of the most prominent political and societal topics in the European Union. Eastern European countries, including Hungary, were impacted by the situation since these countries lie on the continental route from the Middle East to western European countries. Similarly to these countries, in Hungary the leading political discourses labeled asylum seekers as migrants who threaten the ethnically and culturally homogeneous country. The criminalization of the asylum seekers contributed to the blurring of the terms migrant, refugee, and asylum seeker (Bansak et al., 2016; Holmes & Castañeda, 2016; Kallius, Monterescu, & Rajaram, 2016). As an opposition to negative responses, solidarity movements also emerged in order to shelter asylum seekers or help them safely cross the country (Kallius et al., 2016). According to a recent study including 15 European countries, (i) humanitarian concerns, (ii) anti-Muslim sentiments, and (iii) economic reasoning were the key factors in the perception of asylum seekers (Bansak et al., 2016).

These polarized opinions do not exist only in terms of semantic processes, but free associations are sensitive to emotional processes (Bradley et al., 1995; Halberstadt et al., 1995; Joffe & Elsey, 2014; Niedenthal et al., 1999). Thus, affective information can indicate the polarization of opinions and it helps to interpret association relations beyond lexical distance/semantic similarity. By combining affective information on free associations to asylum seekers (i.e., emotional labels) with traditional attitude measurements such as perceived outgroup threat (Kteily, Bruneau, Waytz, & Cotterill, 2015; Schweitzer, Perkoulidis, Krome, Ludlow, & Ryan, 2005; Stephan, Stephan, & Oskamp, 2000), group malleability (Halperin, Russell, Trzesniewski, Gross, & Dweck, 2011), and social dominance orientation (Pratto, Sidanius, Stallworth, & Malle, 1994), we aimed to demonstrate how free associations can reveal polarized opinions, distinguished by their affective content and related attitudes.

## Research goals and validation process

In this study, we aimed to demonstrate that co-occurrence statistic of associations can identify polarized opinions in the perception of asylum seekers. For this reason, we constructed networks from free associations, in which associations were considered to reflect opinions and associations were connected base on their statistical co-occurrences (log likelihood ratio, LLR); thus, we refer to our free association networks as networks of co-occurring opinions (CoOp networks). We constructed such CoOp networks from multiple response free associations to the cue “migrant” in the case of two independent and comprehensive samples in Hungary. Subsequently, we identified modules (densely connected subnetworks) of the CoOp networks.

We hypothesized that frequently co-occurring associations have higher emotional similarity (Hypothesis 1). To test this, respondents were asked to evaluate their own associations with emotion labels. The emotional similarity for every pair of associations was calculated on the basis of the difference in the empirical distributions of their emotional labels. We calculated the correlation between emotional similarity values and co-occurrence connection values applying a permutation method (quadratic assignment procedure; QAP).

We tested the stability of the CoOp networks (Hypothesis 2). First, we aimed to test whether the LLR values were correlated between the two samples (Hypothesis 2a). Second, we aimed to test whether the CoOp networks are more similar to each other—on the basis of normalized mutual information—than a large number of randomized networks (null-models) with similar properties (Hypothesis 2b). Third, we aimed to test whether the exclusion of rare associations increase the stability of our method due to the lower proportion of peripheral associations and the higher proportion of core associations (Hypothesis 2c).

We assumed that the modules of the CoOp network reflect different opinions. Therefore, we statistically compared the attitude values (POT, GM, SDO) of participants whose associations belonged to different modules (Hypothesis 3). We assumed that explicit attitudes toward migrants (POT scores) can differentiate between modules more clearly than abstract construct related to perceived outgroup features (GM and SDO scores).

## Method

### Participants and procedure

For our research purposes, two nationally comprehensive samples of Hungarian participants were recruited. The samples were nationally comprehensive in terms of gender, age, level of education, and type of residence for those Hungarians who use the Internet at least once a week. The participants were selected randomly from an Internet-enabled panel including 15,000 members with the help of a market research company in June 2016 (Sample 1) and in October 2016 (Sample 2). The samples were created with a random stratified sampling method among panelists in the online panel of the market research company with the average response rate 25%. Individuals were removed from the panel if they gave responses too quickly (i.e., without paying attention to their response) and/or had fake (or not used) e-mail addresses.

The final samples comprised *N*_S1_ = 505 and *N*_S2_ = 505 respondents who gave valid answers (Male_S1_ = 247, Female_S1_ = 258; Male_S2_ = 249, Female_S2_ = 256). Hungarians aged between 18 and 60 years in both samples (*M*_S1_ = 40.19 years, *SD*_S1_ = 11.78 years; *M*_S2_ = 39.24 years, *SD*_S2_ = 11.9 years). Regarding the highest level of education, 17.62%/17.82% (Sample 1/Sample 2) of the respondents had primary level of education, 0.4%/0.99% studied in secondary school without graduation, 26.14%/25.74% graduated from secondary school, 6.93%/7.13% studied in higher education and 48.91%/48.32% had higher education degree. Regarding the place of residence, 28.51%/28.71% of the respondents lived in villages, 31.49%/31.88% lived in towns, 21.39%/20.79% lived in county capitals and 18.61%/18.61% lived in the capital city.

The Research Ethics Committee of the local university approved this study. Data were collected via an online questionnaire. Participants were informed that the questionnaire was designed for measuring attitudes toward migrants. No other information was provided about the content and respondents could only see the actual task. All participants provided their written informed consent to participate in this study through a check-box on the online platform. The ethics committee approved this consent procedure. Respondents were assured of their anonymity and as a compensation the market research company drew gift cards among those who participated in the study.

### Measures

#### Multiple response free association task

In this study an associative task was used, based on Abric’s (Abric, 1994, 2003) theoretical underpinnings and on Vergès’s (Vergès & Guimelli, 1994) and on Flament and Rouquette’s (2003) methodological assumptions. In the most of the social representation studies, a multiple response (a.k.a. continuous association task) response is applied with a limited number (three or five) of required associations. This method can reduce association chaining effects and inhibitory effects (De Deyne & Storms, 2008b) that are more prevalent in open-ended association tasks. Furthermore, open-ended association tasks can generate a lower number of average responses than a task with a predefined number of responses (Kinsella et al., 2015).

In the present case, the respondent’s task was to write five words or expressions that comes into their mind regarding the word “migrant.” However, in this study, we did not use the traditional methodology of social representations for identifying the central core and periphery or the density of the representations (Abric, 1994; Orosz & Roland-Lévy, 2013; Flament & Rouquette, 2003). Instead, we used a network analytic method. From the perspective of large-scale semantic network studies, multiple response free association tasks generate strong and weak associations as well (De Deyne & Storms, 2008b). Classical social representation studies and network analytic association studies are closely related in terms of data collection procedure. The strong associations can constitute the central core of the representation and weak associations can belong to periphery (Abric, 1993; De Deyne & Storms, 2008b). This associative task was the first question in the questionnaire for avoiding the influence of prior topic relevant questions.

#### Emotional labeling task

After providing all five of the associations, respondents got back their associations one by one and were asked to provide two emotional labels to each of their own associations. We found that the negative-neutral-positive valence evaluation used in prior similar studies (Orosz & Roland-Lévy, 2013) is too constrained. Furthermore, frequently used affect measures as PANAS cannot be effectively used for the present goals as it included several irrelevant items (e.g., active, strong, alert) and excluded relevant ones (e.g., antipathy, empathy, anger). For this reason we reviewed basic emotion theories (Ekman, 1992; Izard, 2013; Ortony & Turner, 1990; Robinson, 2008) to identify topic-relevant emotional labels. More precisely, the selection of the emotions was largely built on the 10 basic emotions of Izard and the 11 pairs of positive and negative emotion pairs of Robinson. However, in a few cases basic emotions were described with synonyms to fit better to the cue. We used the following 20 emotional labels (differences from the original ones can be seen in parentheses): interest–alarm (anxiety), empathy–contempt, surprise–indifference, hope–fear, gratitude–anger, joy–sadness, calmness–relief (frustration), pride–shame, generosity–envy, and love (sympathy)–hate (antipathy). Respondents could choose any two from the 20 emotional labels for each of their own associations (the labels did not appear as opposites).

#### Perceived outgroup threat (POT)

Perceived threat from asylum seekers were assessed using seven items (Sample 1 *α* = .96, Sample 2 *α* = .96) that were translated from an implementation (Kteily et al., 2015) of the integrated threat theory (Stephan et al., 2000). The POT scale was translated to Hungarian according to protocol (Beaton, Bombardier, Guillemin, & Ferraz, 2000), and it was adopted to the contemporary Hungarian context on the basis of a preliminary study (e.g., “Migrants pose a physical threat to Hungarians”). Responses were made on 5-point Likert-type scales (1 = *strongly disagree*, 5 = *strongly agree*). The higher value indicates higher level of perceived threat from migrants. For further details of this measure see Table S1.

#### Group malleability (GM)

We adopted a 4-item (Sample 1 *α* = .95, Sample 2 *α* = .94) version questionnaire (Halperin et al., 2011) to assess respondents’ implicit assumptions on whether social groups are capable of development. The GM scale was translated to Hungarian according to protocol (Beaton et al., 2000; e.g., “Groups can do things differently, but the important parts of who they are can’t really be changed”). Respondents indicated their level of agreement using a 6-point Liker-type scale (1 = *strongly disagree*, 6 = *strongly agree*). The higher value indicates higher level of agreement with the concept of nondeveloping groups. For further details of this measure, see Table S2.

#### Social dominance orientation (SDO)

The Social Dominance Orientation (Pratto et al., 1994) questionnaire has eight items (Sample 1 *α* = .83, Sample 2 *α* = .83) that measure respondents’ degree of preference for inequality among social groups. The SDO measure was translated to Hungarian according to the protocol (Beaton et al., 2000; e.g., “Some groups of people are simply not the equals of others”). Respondents indicated their level of agreement using a 7-point Liker-type scale (1 = *strongly disagree*, 7 = *strongly agree*). The higher value indicates higher level of preferred inequality among social groups. For further details about this measure, see Table S3.

### Preprocessing of associations

The preprocessing and lemmatization of the associations was carried out by four independent coders. Lemmatization is a linguistic process of grouping inflexions of a word into a single word (lemma) without conjugates. In other words, it is basically the grouping of words with the same stem. In the lemmatization process, two associations were merged in the following cases: (i) they had the same lemma (e.g., “refugee” and “refugees” were merged; Flament & Rouquette, 2003); (ii) they were semantically so close that the English translation could not distinguish between them (e.g., “stain” and “dirt”). Two associations were merged only if the coders could reach to a consensus.

### CoOp network construction

Statistical relations among associations were defined on the basis of their co-occurrences to identify connections. We used log-likelihood ratio (LLR) to assess co-occurrence connections between every possible association pairs (Dunning, 1993). For each possible pair of associations, we calculated the likelihood assuming statistical independence for their co-occurrence over the maximum likelihood of the observed co-occurrence:

$$ \lambda \left(i,j\right)=\frac{L\left({j}_n\cap {i}_n,{i}_n,\frac{j_n}{n}\right)\ast L\left({j}_n\cap \neg {i}_n,\neg {i}_n,\frac{j_n}{n}\right)}{L\left({j}_n\cap {i}_n,{i}_n,\frac{j_n\cap {i}_n}{i_n}\right)\ast L\left({j}_n\cap \neg {i}_n,\neg {i}_n,\frac{j_n\cap \neg {i}_n}{\neg {i}_n}\ \right)}, $$

where *j*_n_ and *i*_n_ denote the number of participants, who mentioned associations *i* and *j*, ¬*i*_*n*_ denotes the number respondents, who did not mentioned association *i* and *n* denotes the number of all participants. L(arg1,arg2,arg3) refer to the probability of a binomail distribution (L) in which *arg1* number of succes occured from *arg2* number of observations and *arg3* is the probability of *arg1*. More genenrally, the above formula measures the level of statistical dependence between *i* and *j* by testing whether the distribution of *j* given that *i* is present is the same as the distribution of *j* given that *i* is not present. The LLR from *λ* is calculated as

$$ LLR\left(i,j\right)=\left\{\begin{array}{c}- ln\lambda \left(i,j\right)\mid \frac{j_n\cap {i}_n}{n}\ge \frac{j_n}{n}\ast \frac{i_n}{n}\\ {} ln\lambda \left(i,j\right)\mid \frac{j_n\cap {i}_n}{n}<\frac{j_n}{n}\ast \frac{i_n}{n}\end{array}\right.. $$

Therefore, the LLR between association *i* and *j* was positive (attractive) if their observed co-occurrence number was higher than the expected one and negative (repulsive) if their observed co-occurrence number was lower than the expected one. Basically LLR performs the same task as χ^2-^test parametric method without the requirement of normality. Multiplication of our LLR values by two can relate them to a χ^2^ distribution with the appropriate degrees of freedom.

We constructed CoOp networks, in which the nodes assigned by the associations and edge weights between nodes determined by LLR values. The nodes were the different associations from the total collections of associations. We ignored associations that occurred fewer than three times, since these are not stable parts in the perception of the social object (Abric, 1993) or possibly are related to idiosyncratic expressions; thus, they do not belong to the social representation (Sarrica, 2007). Furthermore, the removal of these nodes ensured higher robustness of the networks.

### Affective similarity

Every participant chose two emotional labels from among the 20 options described above to characterize their affective relation to each of their association. The affective similarity between every pair of associations was calculated as

$$ \mathrm{Affective}\ \mathrm{similarity}\ \left(i,j\right)=2-{\sum}_{e=1}^{20}\left|\frac{E\left(e,i\right)}{\sum_{e=1}^{20}E\left(e,i\right)}-\frac{E\left(e,j\right)}{\sum_{e=1}^{20}E\left(e,j\right)}\right|, $$

where *i* and *j* are two different associations, *e* is the emotional label (ranging from 1 to 20), and *E* is a two dimensional matrix, in which each item *E*(*e,i)* refer to the number of times *e* emotion assigned by the respondents to association *i*. The similarity value of 2 indicates identical emotional labels, whereas the similarity value of 0 indicates totally different emotional labels between two associations.

### Module detection

Both CoOp networks were divided into nonoverlapping sets of densely linked associations (modules). A modularity maximization process (Newman & Girvan, 2004) was applied to identify the modules of the networks. The original modularity formula is generalized (Gómez, Jensen, & Arenas, 2009) to deal with both the positive (attractive) and negative (repulsive) links:

$$ Q=\frac{1}{v^{+}+{v}^{-}}\sum \limits_{ij}\left[\left({w}_{ij}^{+}+{e}_{ij}^{+}\right)-\left({w}_{ij}^{-}+{e}_{ij}^{-}\right)\right]{\partial}_{M_i{M}_j}, $$

where *Q* denotes the modularity value of a given partition of a network, *v*^+^/*v*^−^ denote the total positive/negative weights of the network, ${w}_{ij}^{+}$/${w}_{ij}^{-}$ denote the positive/negative weights between node *i* and *j*, ${e}_{ij}^{+}$/${e}_{ij}^{-}$ denote the chance-expected positive/negative connections between node *i* and *j*, ${\partial}_{M_i{M}_j}$ is an indicator function that is set to 1 if node *i* and *j* belong to the same module. The higher the modularity of a network partition, the higher the difference between the fraction of edges fall within the modules minus the expected fraction of edges fall within the same modules in a corresponding random network. The Louvain algorithm (Blondel, Guillaume, Lambiotte, & Lefebvre, 2008) was applied to identify the modular partition with the highest possible modularity, namely the highest ratio of edge weights inside the modules and lowest ratio of edge weights between modules. Therefore, the size and number of modules belong to the modular partition with the maximal modularity is parameter-independent and match with the structure of the network. In our case, it is extremely important to determine algorithmically the number of modules (i.e., number of opinion dimensions) that best describe the data. A drawback of modularity maximization that the resulting modular structure can change in each iteration (Good, Montjoye, & Clauset, 2010) as the optimization process may stuck in local maxima. Therefore, a consensus partition was determined for the sake of higher reliability (Lancichinetti & Fortunato, 2012). In the consensus partitioning process, first the consensus matrix was determined on the basis of 5,000 independent iterations of the Louvain algorithm. The edge weights between every pair of nodes in the consensus matrix determined on the basis of the number of times two nodes fall into the same module. The consensus matrix was partitioned to nonoverlapping modules 100 times by the Louvain algorithm. If the resulting 100 partitions were identical, then it was accepted as the consensus partition, otherwise the sets of 100 partitions were generated from the consensus matrix until the agreement. The average modularity score of the 5,000 independent iterations and the consensus partition of the CoOp networks were determined for both samples.

### Reproducibility test

To demonstrate stability regarding the co-occurrences of associations and the identified modular structure, we compared the LLR edges and modular structures of the two independent samples (Sample 1 and Sample 2). Since associations were slightly different in the two samples, only the identical associations were compared in terms of LLR value and modular membership. The similarity of the LLR value between identical associations in the two samples was measured by Spearman’s correlation. The significance of the correlation was determined by QAP (Simpson, 2001). A simple pairwise correlation between the LLR values of the two samples would assume the independence of the edges, however a node in a network typically have similar connections, thus multiple similar edges belonging to one node can cause spurious correlation. QAP is a permutation procedure to eliminate the effect of interdependence between network edges belonging to a common node (Simpson, 2001). First, QAP determined the similarity of the LLR values of the two networks. This was done by Spearman’s correlation in our case. Second, the edges of the CoOp network in Sample 1 were randomly shuffled by permuting the rows and columns of the adjacency matrix in the same order. Third, Spearman’s correlation was calculated between the LLR values of the shuffled CoOp network and the LLR values of the CoOp network from Sample 2. The second and third parts of the QAP were repeated 5,000 times, and the absolute values of the simulated correlation coefficients were saved. The level of significance (*p*_QAP_) was equal to the percentile of the simulated correlation coefficients reached the level of the correlation coefficient from the real data.

The similarity of the modular structures was measured by means of normalized mutual information (nMI):

$$ nMI=2\ast \frac{H(M1)+H(M2)-H\left(M1,M2\right)}{H(M1)+H(M2)}, $$

where *H*(*M*1) and *H*(*M*2) are the entropies of the modular partitions of Sample 1 and Sample 2 separately, and *H*(*M*1,*M*2) is the joint entropy of the two partitions (Meilǎ, 2007). Since 5,000 independent networks were created for both samples to determine the consensus partitions, we determined the final nMI value as the average of all pairwise comparisons of the modular structures based on LLR edge weights.

To determine whether the similarity between modular organizations of the two samples indicates a nonrandom similarity, we compared the nMI calculated from the similarity of the original CoOp networks with the nMI calculated from the similarity of the null models. The simplest null model is the Erdős–Rényi graph, in which the edges are randomly rewired; however, more sophisticated null-model generation procedures can maintain certain parameters of the original network in the random network. Here, we generated edge-, weight-, and strength-preserving random networks (Rubinov & Sporns, 2011) for both Samples 1 and 2. The generation of the null model consisted of two steps. First, the randomization of the network was done by connection-switching method (Wormald, 1999) in a way that preserved the positive and negative degrees of the nodes. Then the weights were allocated and iteratively rearranged to converge to the weight distribution of the original network (Rubinov & Sporns, 2011). A set of 5,000 null models were generated and the modular structures of the null models were determined. The similarity (nMI) of the modular structures (only identical association included) was calculated for the null models. The process resulted in a distribution of nMI values. The observed nMI value was compared to the nMI values derived from the null models. The CoOp networks of Sample 1 and Sample 2 were considered significantly similar if the observed nMI value was higher than 95% of the nMI values derived from the null model comparisons.

To demonstrate that higher numbers of observations offer a higher stability of our method, we iteratively raised the threshold of the ignored associations from the default 3 to 13. The similarity of the LLR edges and modular structures were calculated for each threshold between Samples 1 and 2.

### Statistical analysis

All statistical analyses were performed with MATLAB version R2014b (The MathWorks Inc, Natick, MA). The applied network measures are all available at https://sites.google.com/site/bctnet/ (Rubinov & Sporns, 2010). Differences of the POT scores were determined by an independent *t* test between Samples 1 and 2.

We calculated the correlation with a permutation test based on QAP (Simpson, 2001) to test whether cognitive attraction is related to affective similarity and cognitive repulsion is related to affective dissimilarity. In the QAP procedure, we moderated the effect of near zero co-occurrence connection values. On one hand, many near zero LLR values were expected between associations never mentioned together, but these association pairs could be characterized by very heterogeneous affective similarity values. On the other hand, moderating the effect of the numerous near zero connections can generate a more balanced LLR data for the correlation analysis, in which the low and high LLR values have similar sampling. Hence, co-occurrence connection values were divided into 100 equal intervals in which the values were averaged. This way, the large number of data points representing near zero co-occurrence values were reduced into averages of a few intervals. The affective connection values were averaged for the association pairs that belonged to a given interval of the co-occurrence connection values. All correlation coefficient was calculated between these averaged values.

The final test of our method was to demonstrate that we can differentiate the modules in CoOp networks according to the attitudes toward asylum seekers. Respondent were assigned to the CoOp modules to which the majority of their associations belonged. Respondents were compared by pairwise independent weighted *t* test on their attitude scores between every pair of modules. Weighted attitude score means (WAM) and weighted attitude score variance (WAV) were calculated for each module (*M*) for weighted *t* tests:

$$ {WAM}_M=\frac{\sum \limits_{i\epsilon M}\left({AttitudeScore}_i\ast {AssociationNumber}_i\right)\ }{\sum \limits_{i\epsilon M}\left({AssociationNumber}_i\right)}, $$

$$ {WAV}_M=\frac{\sum_{i\epsilon N}{AssociationNumber}_i\ast {\left({AttitudeScore}_i-{WeightedAttitudeMean}_M\right)}^2}{\sum_{i\epsilon N}{AssociationNumber}_i}, $$

where *i* is a respondent assigned to module M. Attitude scores of a respondent (*AttitudeScore*_*i*_) were weighted equally to the number of their associations that belonged to the given module (AssociationNumber_*i*_). A respondent was discarded from the attitude analysis if he or she could have been assigned to more modules with equally maximum weights. The statistical procedure was conducted on Samples 1 and 2 separately.

Since our study was exploratory, we carried out statistical power estimation for a theoretically medium effect size (Cohen’s *d* = 0.5), which we determined to be the indicator of a considerable opinion difference between the respondents assigned to two given modules. We concluded that .8 power could be achieved if the sample size was 64. (Power was determined for Cohen’s *d* = 0.5 with alpha = .05. In the calculation, normal distributions were assumed, with a mean difference equal to 0.5 and a standard deviation equal to 1.)

## Results

The total numbers of different associations were 1,067, in the case of Sample 1, and 1,099, in the case of Sample 2. After the lemmatization, the numbers of different associations decreased to 597, in the case of Sample 1, and 533, in the case of Sample 2. The numbers of associations mentioned at least three times—and thus that were included in the network analysis—were 156 in the case of Sample 1, and 163 in the case of Sample 2. Samples 1 and 2 had 114 identical associations. Thus, the analysis was performed on 1,966 association tokens in Sample 1 and on 2,023 association tokens in Sample 2. The POT scores showed no significant overall difference between Sample 1 (*M* = 3.33, *SD* = 1.37) and Sample 2 (*M* = 3.43, *SD* = 1.36).

### CoOp connections and affective similarity (Hypothesis 1)

**Fig. 1:**
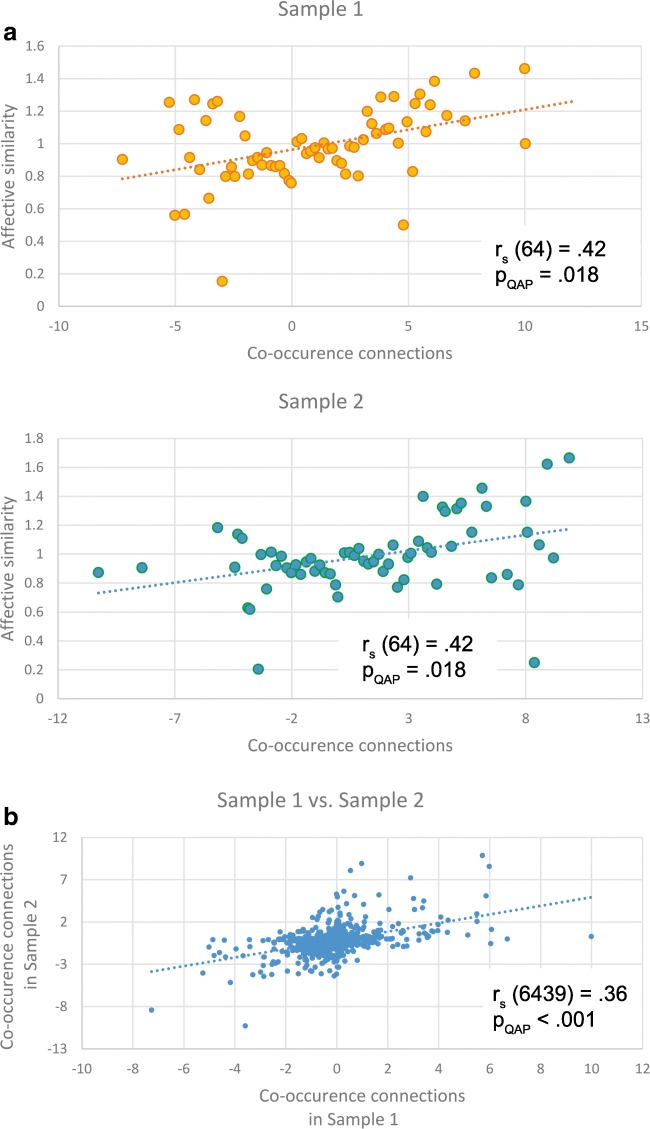
Correlations between affective similarity values and co-occurrence connections (A) and correlations between the identical co-occurrence connections in Samples 1 and 2 (B). (A) The *x*-axes show the co-occurrence connection values, and the *y*-axes show the affective similarity values. The *x*-coordinates of the data points represent averages of the co-occurrence connection values in each of the 100 equal intervals. The association pairs were determined in each interval, and their affective similarity values were also averaged. The *y*-coordinates of the data points represent these averaged affective similarity values. (B) The similarity of the co-occurrence connections between identical associations in the two samples was measured by Spearman’s correlation. The *x*-axis shows the co-occurrence connections in Sample 1, and the *y*-axis shows the co-occurrence connections in Sample 2

Significant correlations were found between the co-occurrence and affective similarity values (Fig. 1) of Sample 1 [*r*_s_(64) = .42, *p*_QAP_ = .018] and Sample 2 [*r*_s_(62) = .39, *p*_QAP_ = .035].

### CoOp modules

**Fig. 2:**
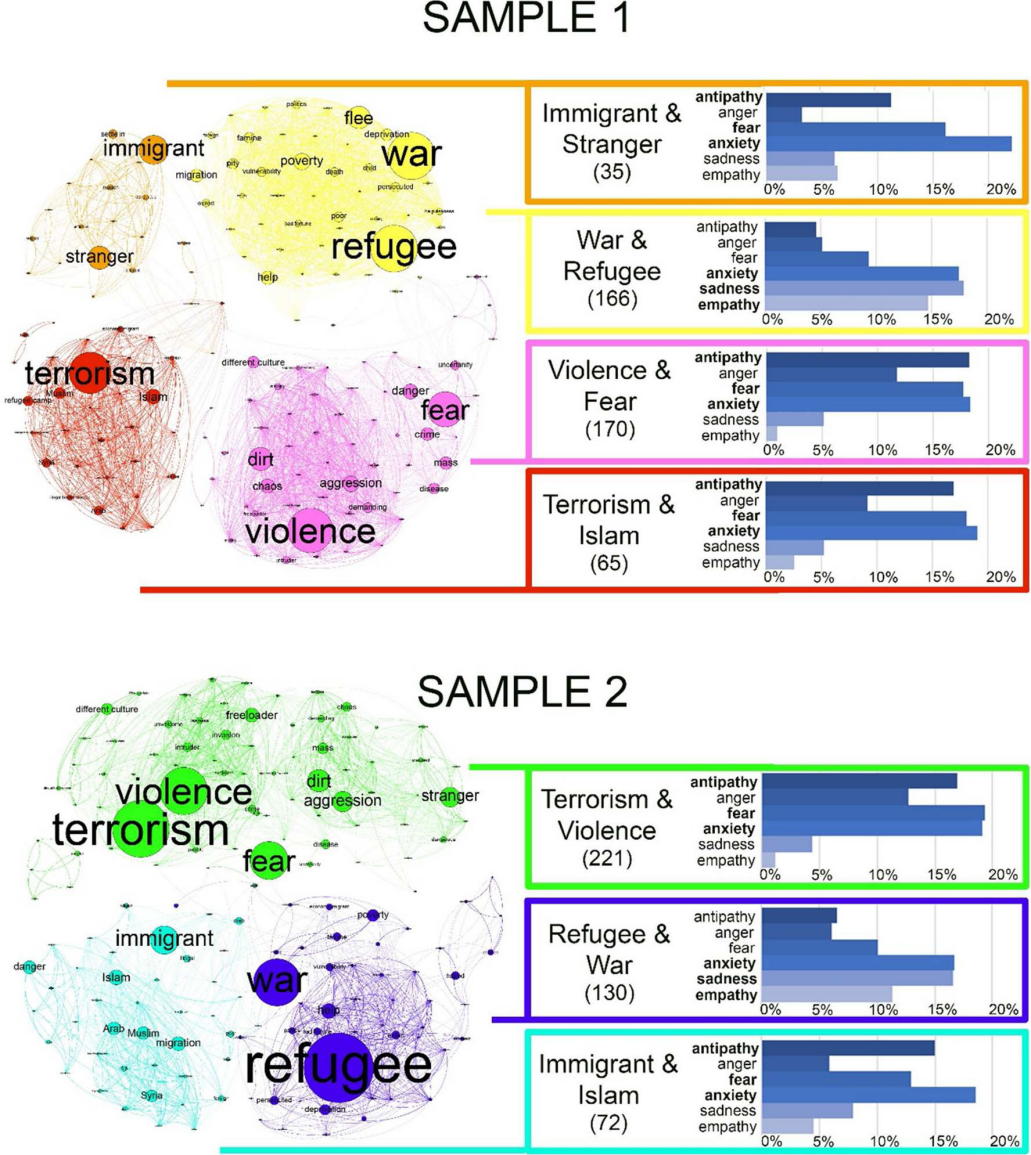
Modules of the CoOp networks. Each module is visualized with different colors. The sizes of a node and its label are proportional to the frequency of the given association. An edge means that two associations fall into a common module in the consensus-partitioning procedure at least 40%. The edges with the LLR edges are presented in Table S6 (Sample 1) and Table S7 (Sample 2). Both samples are displayed by the “Yifan Hu Proportional” layout algorithm (Hu, 2005), implemented in Gephi (Bastian, Heymann, & Jacomy, 2009). Additional information about each module is shown in a box colored identically to the corresponding module. The box contains the label of the module, referring to the two most frequent associations in a given module. The number of respondents assigned to a given module is displayed below the label in parentheses. The percentages of emotional labels for every module are presented on bar charts. The percentages of the six most frequent emotions (antipathy, anger, fear, anxiety, sadness, empathy) are shown in detail. The three most frequent emotions for a module are displayed with bold letters. (For detailed distributions of the affections in every module, see Table S8.)

We labeled the modules according to the two most frequent associations (see Fig. 2). The modular membership and frequency of every association are presented in Table S4 (Sample 1) and Table S5 (Sample 2).

The modularity value was .24 for the CoOp network of Sample 1, and this value was .23 for the CoOp network of Sample 2. The CoOp network of Sample 1 was divided into four modules, and the CoOp network of Sample 2 was divided into six modules. However, in Sample 2, three of the six identified modules contained only a single word, each mentioned by a few respondents (“assassination,” “unity,” and “death”). We did not include these modules in the further analyses, so the final number of modules was three in the case of Sample 2.

### Reproducibility (Hypothesis 2)

To test the reproducibility of our method, we derived an edge-level and a modular-level comparison between Samples 1 and 2. The LLR-level comparison was performed by correlation of the LLR values between the identical association pairs of Samples 1 and 2. We have found a significant correlation between the LLR values of the identical association pairs in Samples 1 and 2 [*r*_s_(6439) = .36, *p*_QAP_ < .001]. The modular-level similarity was determined by the nMI value of the modular membership of the identical associations between Samples 1 and 2. The similarity between the modular structures of the two samples was significantly higher than in the corresponding null models (nMI = .27, *p* < .001). Precisely, none of the 5,000 generated null models had an nMI value higher than the nMI value between Samples 1 and 2.

**Fig. 3:**
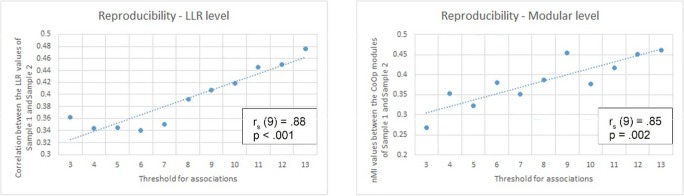
Correlations between the reproducibility and exclusion of rare associations from the analysis. The *x*-axes show the minimal numbers of occurrences of an association. Below that occurrence number, an association was excluded from the analysis. The *y*-axes show the LLR-level (A) and modular-level (B) similarities between Sample 1 and Sample 2. The LLR-level similarity was expressed by the Spearman correlation of the LLR values between the identical association pairs of Samples 1 and 2. The modular-level similarity was determined by the similarity of the modular memberships of Samples 1 and 2. The modular-level similarity was expressed by the nMI value. The exclusion of rare associations resulted in higher LLR similarity (A) and higher modular similarity (B) between Samples 1 and 2

The changes in LLR-level and modular-level similarity between the two samples were determined by ignoring associations that occurred less than a given threshold value. The threshold was iteratively raised from the default of 3 to 13. Strong and significant correlations were detected between the threshold and the LLR-level [*r*_s_(9) = .88, *p* < .001] and modular-level [*r*_s_(9) = .85, *p* = .002] similarities of Sample 1 and Sample 2. Ignoring sparse associations from the analysis could raise the edge- and modular-level similarities between Samples 1 and 2. Details about the edge- and modular-level similarities for every threshold are presented in Fig. 3, Table S9, and Table S10. Here we only present the LLR-level similarity [*r*_s_(559) = .48, *p*_QAP_ < .001] and modular-level similarity (nMI = .46) for the analysis when ignoring associations that occurred fewer than 13 times.

### CoOp modules and POT scores (Hypothesis 3)

**Fig. 4:**
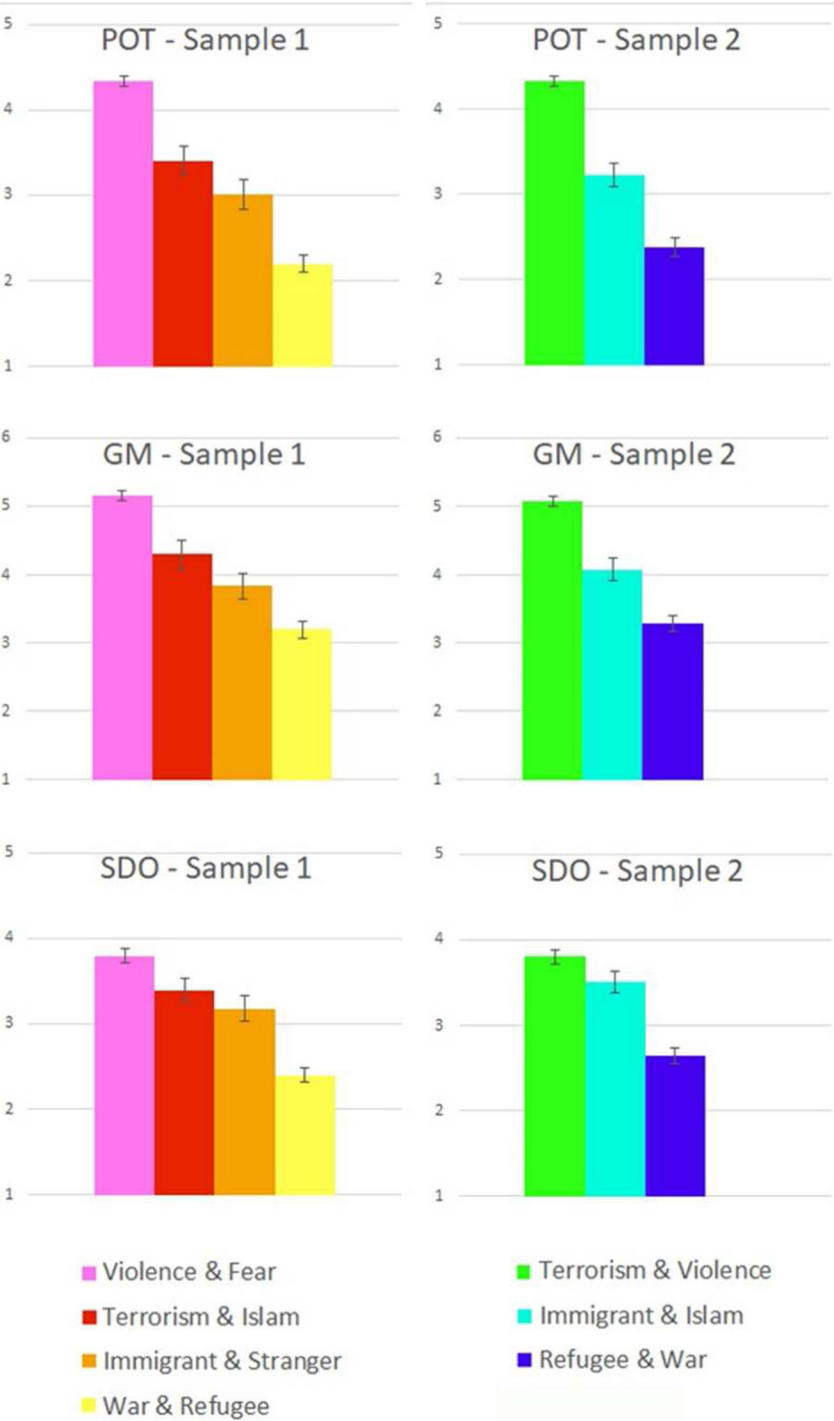
Perceived outgroup threat (POT), group malleability (GM), and social dominance orientation (SDO) scores of the modules in Samples 1 and 2. Bars represent the means of the scores for every module. Standard errors are presented on the bars. All pairwise comparisons of the modules showed significant differences in POT scores. See the detailed POT analysis results. (The GM and SDO results are presented in Tables S11 and S12.)

In the case of Samples 1 and 2, all pairwise comparisons of the modules showed significant differences in POT scores (Fig. 4).

In the case of Sample 1, respondents assigned to the War & Refugee module (*M* = 2.25, *SD* = 1.20) showed significantly lower POT scores than did respondents assigned to the Immigrant & Stranger [*t*(199) = – 3.23, *p* < .001, *d* = 0.57], Terrorism & Islam [*t*(229) = – 6.65, *p* < .001, *d* = 1.01], and Violence & Fear [*t*(334) = – 18.49, *p* < .001, *d* = 2.03] modules. The Immigrant & Stranger module (*M* = 2.93, *SD* = 1.12) had a significantly lower POT score than did the Terrorism & Islam [*t*(98) = – 2.27, *p* = .013, *d* = 0.45] and Violence & Fear [*t*(203) = – 6.91, *p* < .001, *d* = 1.62] modules. The Terrorism & Islam module (*M* = 3.50, *SD* = 1.31) had a significantly lower POT score than did the Violence & Fear module [*t*(233) = – 4.64, *p* < .001, *d* = 0.84], which showed the highest POT score (*M* = 4.30, *SD* = 0.78). In the case of Sample 1, the statistical comparisons involving the Immigrant & Stranger and Terrorism & Islam modules did not have sufficient power.

In the case of Sample 2, respondents assigned to the Refugee & War module (*M* = 2.10, *SD* = 1.21) had significantly lower POT scores than did respondents assigned to the Immigrant & Islam [*t*(200) = – 6.92, *p* < .001, *d* = 0.99] and Terrorism & Violence [*t*(349) = – 18.71, *p* < .001, *d* = 2.27] modules. The Immigrant & Islam module (*M* = 3.25, *SD* = 1.13) had a significantly lower POT score than did the Terrorism & Violence module [*t*(291) = – 7.39, *p* < .001, *d* = 1.17], which showed the highest POT score (*M* = 4.31, *SD* = 0.83). In the case of Sample 2, all comparisons could be considered to have a power of .8.

Similarly to the POT scores, the GM and SDO scores were compared across the modules. Detailed results about the GM and SDO analyses are presented in Tables S11 and S12. In most cases—similarly to POT—these measure could differentiate the modules. Here we only give a short overview about the few exceptions, where we did not get a significant difference or sufficient power. In the case of Sample 1, the comparisons of every module gave significant differences in the GM analysis, but the comparison of Immigrant & Stranger with Terrorism & Islam did not have sufficient power. In the case of Sample 2, all comparisons were significant with sufficient power. In the case of Sample 1, the comparison of the modules in terms of SDO scores failed to detect a significant difference between the Immigrant & Stranger and Terrorism & Islam modules, and the comparison of the Terrorism & Islam and Violence & Fear modules did not have sufficient power. In the case of Sample 2, the comparisons of the modules in terms of SDO scores all produced significant differences, although the comparison of the Immigrant & Islam and Terrorism & Violence modules did not reach sufficient power. In sum, POT, GM, and SDO showed very similar patterns in most of the cases.

## Discussion

In this study, we aimed to introduce and validate a method that identifies groups of associations reflecting distinct attitudes and emotions toward demonstrative cue: migrants. In line with Hypothesis 1, the co-occurrence of the associations (CoOp networks) reflected the emotional similarity between the associations. In line with Hypothesis 2, the modular structures of CoOp networks showed considerable reproducibility in the two independent samples. In line with Hypothesis 3, the distinct cohesive structures of associations (CoOp modules) reflected different results on the POT, GM, and SDO measures. For example, between modules reflecting on *violence* (Violence & Fear, Terrorism & Violence) and *refugee* (War & Refugee, Refugee & War) always demonstrated significant differences in the three measures (POT, GM, and SDO). In sum, the present results demonstrated that analyzing the modular organization of CoOp networks can be an inductive tool for identifying the most important dimensions of public opinions about relevant social issues.

CoOp networks can be seen as a subtype of large-scale semantic networks (De Deyne & Storms, 2008a; Nelson, McEvoy, & Schreiber, 2004; Steyvers & Tenenbaum, 2005). Semantic networks are built from multiple cues and organized by constant lexical relations. Our study demonstrated that co-occurrences of multiple free word associations can also follow affective similarity patterns regarding a social issue. This is in line with cognitive studies on roles that emotions play in mental process—for instance, message acceptance/rejection and information recall (Nabi, 1999, 2003). Our results also highlight that module detection in CoOp networks yields a psychologically meaningful mapping of context behind attitudes. The modular membership of the associations creates a context for the interpretation of each individual association. Furthermore, the jointly interpreted associations can link the attitudes to the relevant context. More generally, consistent patterns in individual association sequences can reveal the most prominent frames of opinions regarding a social issue.

The polarization of opinions was consistent in the two samples with a positive pole indicated by terms such as “Refugee,” “War,” or “Help” and a negative pole indicated by terms such as “Violence,” “Fear,” or “Terrorism.” Furthermore, modules reflecting these poles comprised the majority of all the respondents in both samples. The Violence & Fear (Sample 1) and Terrorism & Violence (Sample 2) modules had the highest POT scores. These modules indicate explicit hostility (Dovidio, Kawakami, & Gaertner, 2002) such as labeling asylum seekers as morally inferior (Haslam & Loughnan, 2014; e.g., “dirt,” “lazy,” “demanding,” “freeloader” associations) or emphasizing perceived threats (e.g., “terrorism,” “crime,” “invasion” associations; Holmes & Castañeda, 2016; Kallius et al., 2016; Stephan et al., 2000). The War & Refugee (Sample 1) and Refugee & War (Sample 2) modules reflect humanitarian concerns and show the lowest POT scores, relative to the other modules. The scores and the contents of these modules indicate that considering asylum seekers as refugees who are forced to leave their homes (e.g., “war,” “famine,” “death,” “flee” associations) is linked to social solidarity (e.g., “help,” “pity” associations) (Appelbaum, 2002; Nickerson & Louis, 2008; Verkuyten, 2004).

As compared to Bansak et al. (2016), we could identify modules referring to (i) humanitarian concerns [the War & Refugee (Sample 1) and Refugee & War (Sample 2) modules] and (ii) anti-Muslim sentiment [the Terrorism & Islam (Sample 1) and Immigrant & Islam (Sample 2) modules], but we did not find modules referring to (iii) economic reasoning. Humanitarian concerns are unequivocally present in Hungarians’ perceptions of asylum seekers, consistent with Bansak et al.’s results. However, our results indicate that general xenophobia and perceived threats are far more salient than economic or religious concerns.

The LLR values between the identical associations of Sample 1 and Sample 2 showed significant correlation and that the CoOp networks referring to relative stability have a modular structure as compared to the null model in a three-month-long interval. The differences between Samples 1 and 2 could have originated in the uncertainty of our method and also in complex influential factors related to the “migration crisis” that occurred in the three months between the collection of Samples 1 and 2 (e.g., the terror event in Nice, a national referendum on immigration, etc.). For example, the association “terrorism” can indicate possible changes in opinions between the two samples. Even before the current asylum seeker situation, “terrorism,” “violence,” and “Islam” were frequently linked by individuals (Ernst-Vintila, Delouvée, & Roland-Lévy, 2011; Sides & Gross, 2013). This is in line with Sample 1, in which “terrorism” belonged with Muslim-related stereotypes (Terrorism & Islam). However, “terrorism” belonged to a module reflecting explicit hostility (Terrorism & Violence) in Sample 2. A possible explanation can be that between the two data gatherings, a significant terror attack happened in France (Nice, in July, 2016; BBC News, 2016), leading to increased securitization discourse of migration in the political media (Holmes & Castañeda, 2016).

Our method also showed higher reproducibility in the case of frequent than of rare associations. From an information theoretical point of view, these results suggest that frequent associations resulted in a more stable pattern of co-occurrences. Following this logic one can reach the desired stability by increasing the sample size. From the social psychological point of view, frequent associations more likely to belong to the core structure referring to a higher stability over time than rare peripheral associations (Abric, 1993; Kinsella et al., 2015). It is possible that complex influential factors such as media can more likely affect the peripheral elements of the representation. This is in line with Abric’s (1993) description of progressive transformation in social representations. In sum, reducing the effect of influential factors and the sparsity of the data by excluding rare associations increased the stability of the results, which suggests the reliability of the applied methodological framework.

The measure on word co-occurrence and the appropriate clustering method were selected on the basis of the following considerations. First, frequency of associations—similarly to word occurrence in a corpus—had a power law function (Zipf, 1935), thus an adequate similarity measure should deal with associations occurring sparsely. The LLR was successfully used in previous text processing designs to measure typical word co-occurrences in large corpus of sentences (Bordag, 2008; Dunning, 1993). In our case, a five-associations-long response sequence was considered as a sentence and the typical pattern of co-occurrence across the sequences was measured by the LLR. The first advantage of LLR that it does not depend on normality as well as it allows the comparison of the co-occurrence of both rare and common associations (Dunning, 1993). Second, the LLR can handle the attraction and repulsion of association pairs based on the expected number of co-occurrences, in the case of independence for two associations. In contrast, a simple co-occurrence count can only distinguish between weak and strong connections. For example, simple co-occurrence count gave a relatively high value (i.e., strong connection) between the Violence and Refugee associations (6/13 in Sample 1/Sample 2) as compared to the other co-occurrence values in our data. However, on the basis of the frequencies of the two associations (93/99 for Violence and 97/146 for Refugee in Sample1/Sample2), expected co-occurrence should have resulted in a higher co-occurrence count (17/28 in Sample 1/Sample 2). The expected co-occurrence was related to the observed co-occurrence count in the LLR formula and resulted in a high negative value (i.e., strong repulsive connections; – 7.27/– 8.4 in Sample 1/Sample 2). Third, LLR can be related to the cumulative distribution of *χ*^2^ test with one degree of freedom, hence one can calculate the significance of the co-occurrences. The modularity-clustering procedure can give a partitioning that matches with the structure of the network without selecting parameters. Most importantly, the size and number of the modules are not predefined (as in K-means clustering) or assigned by the researcher on the basis of a dendrogram (as in Ward’s method). The parameter-free and unconstrained characteristics of the modularity formula ensures the data-driven clustering of associations.

The major limitation is that connections of the CoOp networks were often created from relatively few observations. As a consequence of this sparsity, it is important to be careful with interpretations based on a single connection and to rely more on the modules that were proved to be meaningful indicators of different attitudes. Furthermore, the modular investigation of the CoOp network is as an exploratory analysis. Therefore, a minimum number of respondents cannot be guaranteed in each module. As an example, three modules were identified containing only one association in the case of Sample 2 (“assassination,” “unity,” and “death”). As a consequence, we cannot provide a lower bound (holding for all comparisons) for statistical power. However, small modules can be filtered according to future study designs to achieve a desired statistical power for a given effect size.

We will now provide a few recommendations for further similar studies to choose an appropriate sample, cue and additional questionnaires for the associations. Large and diverse sample is recommended to increase the stability of the method (increased threshold for ignoring associations increase the stability) and to capture the heterogeneity of opinions in the target group. Selection of the appropriate cue for the study is crucial. Most importantly, the respondents should have an elaborated opinion about the provided cue. For example, there should be an active group-level discourse about the topic in the target group. In our case, during data collections migration was a prominent topic in the political public and media discourses for the Hungarian population. Indefinite cues should be avoided; different respondents can easily provide different meanings for a cue, hence the segregation of the CoOp modules can easily reflect to semantic differences. For instance, the cue *play* can refer to *sport*, *music*, or *games* (Lancichinetti, Radicchi, Ramasco, & Fortunato, 2011). An appropriate cue should be a single word. Even for compound words certain respondents may associate to the first word as others to the second word. Further studies can also guide associations by manipulating the instructions. For example, simply asking “climate change” as a cue may be result in a CoOp module structure in which technical terms, beliefs and associations for “climate” are segregated. If one is interested in the different beliefs for climate change, the instruction could be restricted to opinions. For the preprocessing of the associations, automatized lemmatization methods are available in the case of English responses—for instance, Porter’s algorithm (Porter, 1980). For sake of higher reliability, we recommend further studies to apply additional questionnaires to test the relevance of the CoOp modules. Although we demonstrated that only the co-occurrence analysis of associations can yield meaningful results, we only tested and validated for a single cue. On the basis of our results, not only an explicit questionnaire about the cue (POT), but questionnaires measuring more abstract constructs (GM and SDO) can differentiate between CoOp modules. This suggests that a broad spectrum of dependent questionnaires is appropriate for testing the modules. Emotional similarity between associations provided a validation metric for LLR values. However, further studies could use the emotional similarity between associations to construct networks and modules. Applying the label of the associations for a similarity measure can help to link directly associations to certain emotional constructs and also gives a less sparse data than co-occurrence measures. It is also important to emphasize that emotional labeling of the associations can be changed to other appropriate labels (e.g., valence, PANAS, etc.). However, we recommend applying a diverse set of potentially relevant labels to maintain the unrestricted nature of the association task.

Future studies could investigate network topological parameters to determine how in individual associations are distributed across modules. These parameters can link the identified modules to individual response patterns. Studying the relation between individual response patterns and the higher-level structure can relate the group-level opinion dynamics to cognitive processes such as biased assimilation (Lord, Ross, & Lepper, 1979) or socio-psychological differences such as SDO or GM in our case. In future studies, the influence of a social object on association relations can be assessed by comparing these relations to a “resting state” baseline of the mental organization among lexical concepts such as large-scale semantic networks (De Deyne & Storms, 2008a; Nelson et al., 2004; Steyvers & Tenenbaum, 2005). Furthermore, constructing questionnaires from data-driven constructs (CoOp modules) can help to converge theoretical and observed dimensions regarding a social object. For example, as opposed to previous studies that had found an emphasis on economic concerns if respondents’ attention was explicitly directed to them, economic concerns did not appear as a governing factor in free individual opinions about asylum seekers. Cross-cultural studies can also apply CoOp network analysis to study how corresponding social objects vary in different cultures and refine questionnaires according to specific cultures (Hainmueller & Hopkins, 2014).

In sum, traditional questionnaires without an inductive focus can hardly reflect the dynamic contents constituting a social object, although these can form a link between social constructs and actual actions (Abric, 1993). The inductive nature of the CoOp modules can contribute to classification of the changing contents that constitute a social object, and it can provide a data-driven representation of characteristic social frames for a particular time and space.

### Author note

B.F. developed the study concept. G.O., B.B., I.T.-K., Z.K., and B.F. contributed to the study design. All authors contributed to the data preprocessing. B.F. carried out the data analysis. B.F. designed the codes. B.F., Z.K., and A.V. interpreted the result under the supervision of G.O.. Z.K., B.F., A.V., and G.O. drafted the manuscript. I.U., J.G., and A.U. provided critical revisions. All authors approved the final version of the manuscript for submission.The authors declare no conflicts of interest.The present work was supported by KAP16-71047-1.2- ITK. The first author (B.F.) was supported by Hungarian Brain Research Program Grants (Grant Nos. 2017-1.2.1-NKP-2017-00002). The last author (G.O.) was supported by the Hungarian Research Fund (NKFI PD 106027, 116686) and Momentum (0183-13 502). I.U. was supported by Hungarian Brain Research Program Grants (Grant Nos. KTIA_13_NAP-A-I/1 and KTIA-13-NAP-A-IV/1-4,6).

## Electronic supplementary material

ESM 1 (DOCX 13 kb)

ESM 2 (DOCX 13 kb)

ESM 3 (DOCX 13 kb)

ESM 4 (XLSX 11 kb)

ESM 5 (XLSX 11 kb)

ESM 6 (XLSX 362 kb)

ESM 7 (XLSX 388 kb)

ESM 8 (DOCX 14 kb)

ESM 9 (DOCX 15 kb)

ESM 10 (DOCX 14 kb)

ESM 11 (DOCX 15 kb)

ESM 12 (DOCX 15 kb)
