# Supplementary material for: Emergence of polarized opinions from free association networks
Source: Behav Res Methods. 2018 Aug 9;51(1):280–94. doi: 10.3758/s13428-018-1090-z (PMC6420605; doi:10.3758/s13428-018-1090-z)
Supplement: Supplementary file 1 — (DOCX 13 kb) [file 13428_2018_1090_MOESM1_ESM.docx]

Table S1.

*Perceived Outgroup Threat Scale* (Kteily et al., 2015)

Instruction: Show how much you agree with each sentence by selecting a number from 1 to 5 on the scale below.

| 1 | 2 | 3 | 4 | 5 |
| --- | --- | --- | --- | --- |
| strongly  disagree | somewhat  disagree | both agree and disagree | somewhat agree | strongly  agree |

1. Migrants pose a health threat to Hungarians.

2. It makes me worry that a migrant child will steal money from me.

3. Migrants pose a physical threat to Hungarians.

4. Migrants demand aid to such extent that it becomes a serious issue for the Hungarian economy.

5. The more migrant children go to a school, the more dangerous the school becomes to other children.

6. Migrants’ culture poses a threat to the Hungarian values.

7. Migrants have customs which I consider threatening.
