# Supplementary material for: Emergence of polarized opinions from free association networks
Source: Behav Res Methods. 2018 Aug 9;51(1):280–94. doi: 10.3758/s13428-018-1090-z (PMC6420605; doi:10.3758/s13428-018-1090-z)
Supplement: Supplementary file 2 — (DOCX 13 kb) [file 13428_2018_1090_MOESM2_ESM.docx]

Table S2.

*Group Malleability Scale* (Halperin et al., 2011)

Instruction: Show how much you agree with each sentence by selecting a number from 1 to 6 on the scale below.

| 1 | 2 | 3 | 4 | 5 | 6 |
| --- | --- | --- | --- | --- | --- |
| strongly disagree | disagree | somewhat disagree | somewhat agree | agree | strongly agree |

1. As much as I hate to admit it, you can’t teach an old dog new tricks--groups can’t really change their basic characteristics.

2. Groups can do things differently, but the important parts of who they are can't really be changed.

3. Groups that are characterized by violent tendencies will never change their ways.

4. Every group or nation has basic moral values and beliefs that can't be changed significantly.
