# Supplementary material for: Emergence of polarized opinions from free association networks
Source: Behav Res Methods. 2018 Aug 9;51(1):280–94. doi: 10.3758/s13428-018-1090-z (PMC6420605; doi:10.3758/s13428-018-1090-z)
Supplement: Supplementary file 3 — (DOCX 13 kb) [file 13428_2018_1090_MOESM3_ESM.docx]

Table S3.

*Social Dominance Orientation Scale* (Pratto et al., 1994)

Instruction: Show how much you favor or oppose each idea below by selecting a number from 1 to 7 on the scale below. You can work quickly; your first feeling is generally best.

| 1 | 2 | 3 | 4 | 5 | 6 | 7 |
| --- | --- | --- | --- | --- | --- | --- |
| strongly oppose | somewhat oppose | slightly oppose | neutral | slightly favor | somewhat favor | strongly favor |

1. An ideal society requires some groups to be on top and others to be on the bottom.

2. Some groups of people are simply inferior to other groups.

3. No one group should dominate in society.

4. Groups at the bottom are just as deserving as groups at the top.

5. Group equality should not be our primary goal.

6. It is unjust to try to make groups equal.

7. We should do what we can to equalize conditions for different groups.

8. We should work to give all groups an equal chance to succeed.
