# Supplementary material for: Emergence of polarized opinions from free association networks
Source: Behav Res Methods. 2018 Aug 9;51(1):280–94. doi: 10.3758/s13428-018-1090-z (PMC6420605; doi:10.3758/s13428-018-1090-z)
Supplement: Supplementary file 8 — (DOCX 14 kb) [file 13428_2018_1090_MOESM8_ESM.docx]

Table S8.

*Emotional labels in Sample 1 and Sample 2.*

|  | ***Sample 1*** | | | | **Sample 2** | | |
| --- | --- | --- | --- | --- | --- | --- | --- |
|  | ***Violence & Fear*** | ***Terrorism & Islam*** | ***Immigrant & Stranger*** | ***War & Refugee*** | ***Terrorism & Violence*** | ***Immigrant & Islam*** | ***Refugee & War*** |
| interest | 0.910364 | 2.849003 | 9.533898 | 2.826087 | 1.658031 | 6.807512 | 2.165507 |
| antipathy | 18.20728 | 16.95157 | 11.22881 | 4.637681 | 16.94301 | 15.02347 | 6.573859 |
| hope | 1.190476 | 2.136752 | 4.661017 | 4.637681 | 1.450777 | 3.286385 | 5.645785 |
| shame | 4.761905 | 4.131054 | 1.059322 | 6.666667 | 3.57513 | 3.286385 | 7.811292 |
| joy | 0.35014 | 0.569801 | 0 | 0.362319 | 0.103627 | 0.352113 | 0.464037 |
| envy | 0.140056 | 0 | 0.211864 | 0.072464 | 0.051813 | 0.469484 | 0.154679 |
| sympathy | 0.420168 | 0.712251 | 1.483051 | 4.202899 | 0.207254 | 1.408451 | 3.557618 |
| indifference | 1.470588 | 2.706553 | 3.177966 | 0.942029 | 0.984456 | 3.99061 | 1.62413 |
| contempt | 9.733894 | 6.837607 | 2.330508 | 2.391304 | 8.186528 | 3.99061 | 3.402939 |
| calmness | 0.280112 | 0.997151 | 1.059322 | 0.652174 | 0.15544 | 1.525822 | 0.618716 |
| sadness | 5.182073 | 5.270655 | 6.144068 | 17.89855 | 4.404145 | 7.86385 | 16.628 |
| empathy | 0.980392 | 2.564103 | 6.355932 | 14.71014 | 1.19171 | 4.460094 | 11.29157 |
| anxiety | 18.34734 | 19.08832 | 22.0339 | 17.46377 | 19.06736 | 18.5446 | 16.70534 |
| gratitude | 0.070028 | 0 | 0.423729 | 0.507246 | 0.103627 | 0.352113 | 0.232019 |
| anger | 11.76471 | 9.116809 | 3.177966 | 5.144928 | 12.6943 | 5.868545 | 6.032483 |
| surprise | 0.560224 | 1.139601 | 3.177966 | 0.724638 | 0.984456 | 1.525822 | 0.773395 |
| fear | 17.71709 | 18.09117 | 16.10169 | 9.347826 | 19.32642 | 12.9108 | 10.13148 |
| pride | 0 | 0.2849 | 0.635593 | 0.289855 | 0.310881 | 0.821596 | 0.309358 |
| frustration | 7.633053 | 6.410256 | 6.144068 | 3.405797 | 8.341969 | 6.57277 | 2.474865 |
| generosity | 0.280112 | 0.14245 | 1.059322 | 3.115942 | 0.259067 | 0.938967 | 3.402939 |

*Note*. The percentages of emotional labels for every module.
