# Supplementary material for: Emergence of polarized opinions from free association networks
Source: Behav Res Methods. 2018 Aug 9;51(1):280–94. doi: 10.3758/s13428-018-1090-z (PMC6420605; doi:10.3758/s13428-018-1090-z)
Supplement: Supplementary file 9 — (DOCX 15 kb) [file 13428_2018_1090_MOESM9_ESM.docx]

Table S9.

*LLR level similarity between Sample 1 and Sample 2 were determined by ignoring rare associations.*

| **Threshold for ignoring associations** | **Spearman's correlation coefficient** | **degrees of freedom** | **p-value for the QAP** | **Total number of associations in Sample 1** | **Total number of associations in Sample 2** | **Total number of identical associations in Sample 1 and Sample 2** |
| --- | --- | --- | --- | --- | --- | --- |
| 3 | 0.36 | 6439 | <.001 | 156 | 163 | 114 |
| 4 | 0.34 | 3568 | <.001 | 123 | 126 | 85 |
| 5 | 0.34 | 2699 | <.001 | 99 | 102 | 74 |
| 6 | 0.34 | 1951 | <.001 | 86 | 85 | 63 |
| 7 | 0.36 | 1651 | <.001 | 73 | 77 | 58 |
| 8 | 0.39 | 1174 | <.001 | 64 | 65 | 49 |
| 9 | 0.41 | 901 | <.001 | 56 | 57 | 43 |
| 10 | 0.42 | 778 | <.001 | 51 | 51 | 40 |
| 11 | 0.45 | 701 | <.001 | 48 | 47 | 38 |
| 12 | 0.45 | 628 | <.001 | 45 | 47 | 36 |
| 13 | 0.48 | 559 | <.001 | 43 | 42 | 34 |

*Note*. Every row of the table contains detailed information about the LLR level similarity of the two sample with different threshold for ignoring associations. Minimal number of occurrence of an association were determined, below that occurrence number, an association was excluded from the analysis (*threshold for ignoring associations*). The similarity of the LLR value between identical association pairs in the two samples was measured by Spearman’s correlation.
