# Supplementary material for: Emergence of polarized opinions from free association networks
Source: Behav Res Methods. 2018 Aug 9;51(1):280–94. doi: 10.3758/s13428-018-1090-z (PMC6420605; doi:10.3758/s13428-018-1090-z)
Supplement: Supplementary file 10 — (DOCX 14 kb) [file 13428_2018_1090_MOESM10_ESM.docx]

Table S10.

*Modular level similarity between Sample 1 and Sample 2 were determined by ignoring rare associations.*

| **Threshold for ignoring associations** | **Normalized mutual information (nMI)** | **Total number of associations in Sample 1** | **Total number of associations in Sample 2** | **Total number of identical associations in Sample 1 and Sample 2** |
| --- | --- | --- | --- | --- |
| 3 | .27 | 156 | 163 | 114 |
| 4 | .35 | 123 | 126 | 85 |
| 5 | .32 | 99 | 102 | 74 |
| 6 | .38 | 86 | 85 | 63 |
| 7 | .35 | 73 | 77 | 58 |
| 8 | .39 | 64 | 65 | 49 |
| 9 | .45 | 56 | 57 | 43 |
| 10 | .38 | 51 | 51 | 40 |
| 11 | .42 | 48 | 47 | 38 |
| 12 | .45 | 45 | 47 | 36 |
| 13 | .46 | 43 | 42 | 34 |

*Note*. Every row of the table contains detailed information about the modular level similarity of the two sample with different threshold for ignoring associations. Minimal number of occurrence of an association were determined, below that occurrence number, an association was excluded from the analysis (*threshold for ignoring associations*). The similarity of the modules membership of the identical association pairs in the two samples was measured by normalized mutual information (nMI).
