# Supplementary material for: Emergence of polarized opinions from free association networks
Source: Behav Res Methods. 2018 Aug 9;51(1):280–94. doi: 10.3758/s13428-018-1090-z (PMC6420605; doi:10.3758/s13428-018-1090-z)
Supplement: Supplementary file 11 — (DOCX 15 kb) [file 13428_2018_1090_MOESM11_ESM.docx]

Table S11.

*Perceived Outgroup Threat, Group Malleability and Social Dominance Orientation scores of the modules in Sample 1 and Sample 2.*

|  | **POT** | **GM** | **SDO** |
| --- | --- | --- | --- |
| **Sample 1** |  |  |  |
| Violence & Fear | 4.30 (0.78) | 5.16 (0.96) | 3.78 (1.01) |
| Terrorism & Islam | 3.50 (1.31) | 4.34 (1.49) | 3.39 (1.07) |
| Immigrant & Stranger | 2.93 (1.12) | 3.77 (1.32) | 3.18 (1.03) |
| War & Refugee | 2.25 (1.20) | 3.23 (1.48) | 2.46 (1.06) |
| **Sample 2** |  |  |  |
| Terrorism & Violence | 4.31 (0.83) | 5.05 (1.07) | 3.82 (1.15) |
| Immigrant & Islam | 3.25 (1.13) | 4.11 (1.4) | 3.52 (1.03) |
| Refugee & War | 2.07 (1.21) | 2.99 (1.35) | 2.44 (1.01) |

*Note*. POT = Perceived Outgroup Threat, GM = Group Malleability and SDO = Social Dominance Orientation scores for every module. Mean and standard deviation (between brackets) were presented for every module.
