# Supplementary material for: Emergence of polarized opinions from free association networks
Source: Behav Res Methods. 2018 Aug 9;51(1):280–94. doi: 10.3758/s13428-018-1090-z (PMC6420605; doi:10.3758/s13428-018-1090-z)
Supplement: Supplementary file 12 — (DOCX 15 kb) [file 13428_2018_1090_MOESM12_ESM.docx]

Table S12.

*Statistical details of all pairwise comparisons of the modules in Sample 1 and Sample 2.*

|  | **POT** | **GM** | **SDO** |
| --- | --- | --- | --- |
| **Sample 1** |  |  |  |
| War & Refugee vs. Immigrant & Stranger | t(199)=-3.23, p<.001,  d=0.57 | t(199)=-2.16, p=.016,  d=0.37 | t(199)=-3.76, p<.001,  d=0.69 |
| War & Refugee vs. Terrorism & Islam | t(229)=-6.65, p<.001,  d=1.01 | t(229)=-5.13, p<.001,  d=0.75 | t(229)=-5.99, p<.001,  d=0.88 |
| War & Refugee vs. Violence & Fear | t(334)=-18.49, p<.001,  d=2.03 | t(334)=-14.14, p<.001,  d=1.55 | t(334)=-11.70, p<.001,  d=1.28 |
| Immigrant & Stranger vs. Terrorism & Islam | t(98)=-2.27, p=.013,  d=0.45 | t(98)=-1.97, p=.026,  d=0.40 | t(98)=-0.95, p=.17,  d=0.20 |
| Immigrant & Stranger vs. Violence & Fear | t(203)=-6.91, p<.001,  d=1.62 | t(203)=-5.89, p<.001,  d=1.35 | t(203)=-3.13, p=.001,  d=0.59 |
| Terrorism & Islam vs. Violence & Fear | t(233)=-4.64, p<.001,  d=0.84 | t(233)=-4.10, p<.001,  d=0.72 | t(233)=-2.53, p=.006,  d=0.38 |
| **Sample 2** |  |  |  |
| Refugee & War vs. Immigrant & Islam | t(200)=-6.92, p<.001,  d=1.00 | t(200)=-5.54, p<.001,  d=0.82 | t(200)=-7.10, p<.001,  d=1.05 |
| Refugee & War vs. Terrorism & Violence | t(349)=-18.71, p<.001,  d=2.27 | t(349)=-14.84,  p<.001,  d=1.74 | t(349)=-11.70, p<.001,  d=1.25 |
| Immigrant & Islam vs. Terrorism & Violence | t(291)=-7.39, p<.001,  d=1.17 | t(291)=-5.20, p<.001,  d=0.81 | t(291)=-2.12, p=.018,  d=0.27 |

*Note*. The statistical details of all pairwise comparisons of the modules’ POT, GM, and SDO scores in Sample 1 and Sample 2. Weighted T-test was applied for the comparisons.
